# Feeding and Development of the Glassy-Winged Sharpshooter, *Homalodisca vitripennis*, on Australian Native Plant Species and Implications for Australian Biosecurity

**DOI:** 10.1371/journal.pone.0090410

**Published:** 2014-03-10

**Authors:** Anna A. Rathé, Leigh J. Pilkington, Mark S. Hoddle, Lorraine J. Spohr, Matthew P. Daugherty, Geoff M. Gurr

**Affiliations:** 1 Plant Biosecurity Cooperative Research Centre, Bruce, Australian Capital Territory, Australia; 2 Graham Centre for Agricultural Innovation (NSW Department of Primary Industries and Charles Sturt University) Central Coast Primary Industries Centre, NSW Department of Primary Industries, Gosford, New South Wales, Australia; 3 Graham Centre for Agricultural Innovation (NSW Department of Primary Industries and Charles Sturt University), Charles Sturt University, Orange, New South Wales, Australia; 4 Department of Entomology, University of California Riverside, Riverside, California, United States of America; 5 NSW Department of Primary Industries, Central Coast Primary Industries Centre, Gosford, New South Wales, Australia; New Mexico State University, United States of America

## Abstract

In any insect invasion the presence or absence of suitable food and oviposition hosts in the invaded range is a key factor determining establishment success. The glassy-winged sharpshooter, *Homalodisca vitripennis*, is an important insect vector of the xylem-limited bacterial plant pathogen, *Xylella fastidiosa*, which causes disease in numerous host plants including food and feedstock crops, ornamentals and weeds. Both the pathogen and the vector are native to the Americas and are considered to be highly invasive. Neither has been detected in Australia. Twelve Australian native plant species present in the USA were observed over two years for suitability as *H. vitripennis* feeding, oviposition and nymph development hosts. Hosts providing evidence of adult or nymph presence were *Leptospermum laevigatum*, *Acacia cowleana*, *Eremophila divaricata*, *Eucalyptus wandoo*, *Hakea laurina*, *Melaleuca laterita* and *Swainsona galegifolia*. An oviposition-suitability field study was conducted with citrus, a favoured oviposition host, as a positive control. Citrus and *L. laevigatum*, *A. cowleana*, *B. ericifolia*×*B. spinulosa*, *C. pulchella*, *E. divaricata*, *E. wandoo*, *H. laurina*, and *S. galegifolia* were found to be oviposition hosts. Egg parasitism by the mymarid parasitoid *Gonatocerus ashmeadi* was observed on all Australian plants. A number of Australian plants that may facilitate *H. vitripennis* invasion have been identified and categorised as ‘high risk’ due to their ability to support all three life stages (egg, nymph and adult) of the insect in the field (*L. laevigatum*, *A. cowleana*, *E. divaricata*, *H. laurina*, and *S. galegifolia*). The implications of these host status and natural enemy research findings are discussed and placed in an Australian invasion context.

## Introduction

*Homalodisca vitripennis* (Germar) (Hemiptera: Cicadellidae) is an invasive pest that has expanded its geographical range significantly, in part due to having a polyphagous feeding habit. *Homalodisca vitripennis* feeds on over 100 plant species from 31 families including exotic crop, ornamental, and native American species [1], [2], [3], [4]. This pest transmits the plant pathogenic bacterium, *Xylella fastidiosa*, which causes disease in a range of crops including grapes, peach and citrus [5].

*Homalodisca vitripennis* lay multiple eggs singly to form egg masses under the epidermis on the abaxial surface. Once hatched, nymphs complete four moults before progressing through five instars over seven to 12 weeks to become highly-mobile winged adults which have a lifespan of several months [1], [6]. *Homalodisca vitripennis* oviposition has been documented twice a year in California, once in early spring (April), and again in mid/summer (June) [1]. *Homalodisca vitripennis* feeds on xylem sap, a nutritionally-poor resource [7], [8], although this is counteracted to some degree by a mutualistic relationship with symbiotic bacteria such as *Baumannia cicadellinicola* and *Sulcia muelleri* that have co-evolved with sharpshooters and provide access to vitamins and amino acids [9]. The advantage of feeding on xylem fluid is the lack of repellents, digestive inhibitors, and toxins. Insects can then expend more energy on extracting the food than detoxifying it [10], [11] and are able to utilize many different hosts as there are no phyto-toxins to overcome. Xylem quality varies with the time of day, season, fertilization, and irrigation [12], [13]. The ability of *H. vitripennis* to use many plant species as hosts for reproduction, development, and feeding allows the insect to cope with diurnal and seasonal changes in xylem fluid [14]. In greenhouse studies, it has been shown that, although possible [15], *H. vitripennis* rarely persists on just one host plant species, and that mortality occurs if caged on a sub-optimal host [14]. The insect may feed on a single sub-optimal species for short periods of time if the energy expended in taking up and metabolising food is not exceeded by the energy in the xylem fluid [13], [14], [16].

Host requirements differ between *H. vitripennis* life stages with adults and fourth and fifth instar nymphs preferring hosts with high amide content in the xylem fluid [8], [14]. Juvenile *H. vitripennis* require a balanced amino acid profile so that essential amino acids (those the insect cannot synthesize) are obtained [8]. *Homalodisca vitripennis* is, therefore, not simply a facultative generalist feeder but utilizes many host species by necessity: a trait which is beneficial for survival in invaded areas devoid of plants in the insects evolved host plant range.

Reproductive success and maintenance of an invasive pest population is determined by the availability of suitable oviposition hosts in the invaded range. *Homalodisca vitripennis* adults tend to oviposit on a range of species rather than just one, but oviposition does not occur on the full range of species that the insect feeds on [14] which is thought to be due to the feeding requirements of nymphs differing from those of adults [13]. In a biosecurity context, information on plant species, especially natives, that can support oviposition by a likely invader, such as *H. vitripennis*, is important because it can inform surveillance programs and allow targeting of control efforts in the event of an incursion.

Natural enemies of *H. vitripennis* include egg parasitoids [17] such as *Gonatocerus ashmeadi* Girault, *Gonatocerus triguttatus* Girault, *Gonatocerus morilli* (Howard) and *Gonatocerus fasciatus* Girault (all Hymenoptera: Mymaridae) [5]. Introductions to California and French Polynesia have proven effective in reducing invasive *H. vitripennis* populations in these locations [18]. Parasitoids locate hosts by using the chemical volatiles produced by some plants in response to *H. vitripennis* feeding as cues. *Gonatocerus ashmeadi* was shown to be attracted to *H. vitripennis*-infested lemon and grapevine plants over 60% more often than those without *H. vitripennis* infestations. However, infested and uninfested *Lagerstroemia indica* L. (which also supports oviposition) were chosen equally as often [19]. This suggests that some *H. vitripennis* host plants may provide an advantage to *H. vitripennis* by facilitating escape from parasitism [19], resulting in ‘enemy free space’. Species that fail to exhibit an induced response to *H. vitripennis* with a subsequent release of kairomones need to be identified as they would favour the invasion process, at least in areas where potential natural enemies exist.

Determining factors such as host plant range of potential invading species for biosecurity assessment is challenging because expensive quarantine facilities and administrative clearance for the importation of the species are required. The alternative is to study the invading species *in situ*, assessing the response of host plant species in their exotic location. Many Australian native plants are widely grown in climatologically similar Southern California, part of the invaded range of *H. vitripennis*. This allowed a two year field study in Riverside, California, USA, to assess the ability of Australian native plants to support feeding, oviposition and nymph development of this biosecurity risk to Australia. The first objective was to determine the relative suitability of select Australian native plants as feeding hosts for *H. vitripennis* or as hosts on which it can complete development. This was assessed via both a field study and a no-choice greenhouse study. The second objective was to quantify the level of *H. vitripennis* egg parasitism on Australian native plants by mymarid parasitoids in the field. Results of these studies are presented here.

## Materials and Methods

### Field site

#### Description of field site and trial

Three field zones were selected at the University of California (zone A: 33.9731, −117.3455, zone B: 33.9711, −117.3417, zone C: 33.9703, −117.3409). Each zone had mature stands of citrus, a favoured *H. vitripennis* host, on three sides to promote population pressure by this pest on study plants. Twelve Australian native plant species were sourced from a commercial nursery in California and were between 0.2 and 0.8 m tall, each in a standard 3.7 L plant pot with medium containing pumice for drainage. Test species used were: family Myrtaceae *Leptospermum laevigatum* (Gaertn) F. Muell., *Callistemon viminalis* (Gaertn.) G. Don, *Eucalyptus wandoo* Blakely, *Melaleuca lateritia* A. Dietr.; family Fabaceae *Acacia cowleana* Tate, *Swainsona galegifolia* (Andrews) R. Br; family Proteaceae *Banksia ericifolia×B. spinulosa* L. f (hereafter “*Banksia* hybrid”), *Grevillea lanigera* A. Cunn. ex Meisn., *Hakea laurina* R. Br.; family Rutaceae *Correa pulchella* Lindl.; family Scrophulariaceae *Eremophila divaricata* (F.Muell.) F. Muell. and family Lamiaceae *Prostanthera ovalifolia* R. Br. Each species was represented according to a randomised block experimental design once in each of four blocks in each of the three zones giving a total of 12 blocks. Nine blocks (3 plants per zone) were surveyed each week for abundance of *H. vitripennis* while three blocks (1 per zone) were surveyed for parasitism. Plants were planted in February 2010 with even spacing between each plant and allowed to establish before the study commenced in June 2010. There was bare earth between all plants and they were irrigated throughout the study receiving water to field capacity one day a week for 12 hours via flood irrigation. Weeds were hand removed weekly.

#### Adult, nymph and egg host plant surveys

Sampling for *H. vitripennis* was carried out weekly in 2010 from June 2 until November 17 and again from June 8 until November 23 in 2011. Nine replicates of each plant species were surveyed each week for *H. vitripennis* abundance (adults and nymphs) and egg masses. On the day of sampling, the whole plant was inspected without touching the plant so as not to disturb insects during the adult and nymph counts. The number of adults and nymphs was recorded during a two minute search period. Branches were then turned over and the number of unhatched egg masses on leaves recorded. Sampling was completed on the same day each week and all plants were surveyed beginning at sunrise before the temperature rose and insects became active. For each of the nine experimental units the total number of *H. vitripennis* over the entire sampling period (25 weeks) was calculated for each year. The same ten randomly selected lemon (citrus) control plants in the groves surrounding the three experimental zones were also sampled in the same manner every two weeks. Control citrus plants were not placed amongst test plants in case they were favoured so strongly that *H. vitripennis* within the zone aggregated on them.

As nymph numbers on study plants were found to be much lower than the number of egg masses, two types of sticky traps were deployed for one week to determine whether hatched nymphs were leaving plants. Yellow sticky cards (23 cm×18 cm, Seabright Laboratories, Emeryville) were attached under plants that exhibited high numbers of hatched egg masses. After one week, cards were collected and examined for nymphs under a dissecting microscope. This was repeated with sheets of transparent plastic coated with Vaseline® in case the yellow colour affected nymph migration. Nymphs were identified as early (instar 1 and 2) or late (instars 3, 4 and 5) stage although for the data analysis, the two stages were pooled due to the low counts.

#### Parasitism on Australian host plants

Parasitoid surveys were conducted on one plant from each species in each of the three zones. This plant was randomly selected, flagged, and excluded from *H. vitripennis* surveys. The plants for the parasitoid surveys were located amongst *H. vitripennis* survey plants so that any volatiles that may have resulted from removing leaves (mechanical plant damage) were not concentrated. Up to two leaves containing intact *H. vitripennis* egg masses were removed from each plant weekly, if present, placed in labelled zip lock bags and transported to the laboratory within an hour of the morning collection. Egg masses were placed in labelled 60 mm Petri dishes (Sigma-Aldrich, P5481) containing moist filter paper and sealed with parafilm to maintain humidity. Insects that emerged from egg masses (either *H. vitripennis* nymphs or parasitoids) were identified to species using a dissecting microscope and counted. Any egg masses that did not hatch after two weeks were dissected under the microscope and the number of un-emerged *H. vitripennis* nymphs and parasitoids was noted. Proportion parasitism was represented by the number of parasitoids to *H. vitripennis* eggs. Since not all plant species had two egg masses per week to sample, the mean proportion of parasitism with 95% confidence intervals was calculated across a different number of egg masses for each plant species per year.

### Greenhouse studies

#### No choice greenhouse feeding study

Feeding rate studies were conducted on eight occasions: four months in 2010 (June, September, October and November) and again over the same months in 2011. Six potted plants of each of the 12 Australian native plant species of interest and a grapevine control plant (*Vitis vinifera* cv Zinfandel, a preferred host) each planted in individual 3.7 L pots were sourced from a commercial nursery and placed in the greenhouse in a 6 replicate randomised block design. New plants were sourced for a repeat of the experiment in 2011. Adult *H. vitripennis* were field collected from a neutral host (citrus) using sweep nets at dawn by gently beating young mandarin trees located on Agricultural Operations land at the University of California, Riverside and placed into 47.5×47.5×47.5 cm insect cages (BD44545F, MegaView Science, Taiwan) containing citrus leaves for transportation. Insects were then mouth aspirated and transferred into new cages containing basil plants (*Ocimum basilicum* L.) in a greenhouse where they were maintained until needed for experiments.

Colony insects maintained on basil were caged individually on experimental plants in 50 ml graduated plastic tubes covered with a layer of 1 mm grid fabric mesh. One male and one female were used per plant as feeding rates can differ based on sex [20]. Two tubes were taped onto each plant flat against the petiole so that the insect had access to a portion of stem through the mesh. Feeding rate was represented by the cumulative total of excreta (ml) in the tube measured via the graduations on the side of the tube every 24 hours for three days. The amount of excreta in each of the two tubes on a plant was averaged to give a single figure per plant and the total over the three days was calculated. Daily insect mortality was also noted and dead insects were not replaced. Plants were watered daily via drip irrigation for 10 minutes. The greenhouse temperature was 25°C (±11°C) which was within a preferred temperature range for excreta production [21].

#### Nymph development greenhouse study

The same plant species as used in the feeding rate study above were used in nymph development greenhouse studies with six replicates. Newly hatched first instar nymphs (<48 hr old) from the colony maintained on basil were placed into a single 2 L plastic bottle cage [22] on each plant with 1 mm grid mesh walls on the sides. Each cage contained two nymphs with access to an entire branch within the cage. Nymphs were monitored daily until death or eclosion occurred [13]. Nymph death was only recorded if a cadaver was recovered, therefore collected data included only those insects that survived to adulthood or had a recorded date of death. This study was repeated three times, in July and August 2010, and July 2011. Plants were watered every second day via drippers for 10 minutes. The greenhouse was maintained at 24°C (±10°C) under ambient light and day length for the duration of the study.

### Statistical methods

#### Adult, nymph and egg host plant surveys

The effect of plant species on season total *H. vitripennis* adult counts was tested using ANOVA in the R programming environment [23] separately for each year of the survey. Species with no *H. vitripennis* observed in all blocks were omitted from analysis. A log_e_ transformation of the total count+1 was necessary to satisfy the variance homogeneity assumption. Species mean counts were separated using the least significant difference (l.s.d.) procedure on the log_e_ scale then back-transformed for presentation here. Similar analyses were conducted for nymphs and egg mass counts. All significance tests were conducted at the α = 0.05 level. Means and standard errors (s.e.) were calculated for adults, nymphs, and egg masses on citrus control plants since they were not part of the randomised experimental design.

#### No choice greenhouse feeding study

The effect of plant species on feeding rate (i.e., excreta production) was tested using a linear mixed-effects model with a square root transformation to satisfy variance homogeneity assumptions. A generalised linear mixed-effects model with binomial error distribution and logit link function was used to test plant species effects on the proportion of insects surviving after 72 hours. The asreml package in R [24] was used for all analyses. Separate analyses were conducted for each year, with random effects of month, replicate and month x replicate included in the models for excreta production and insect survival. Plant species effects were tested with an approximate F statistic at the 5% significance level. Predicted species means were compared on the transformed scale (square root for excreta production; logit for survival) using the l.s.d. technique at the 5% significance level and back-transformed for presentation here.

## Results

### Adult, nymph and egg host plant surveys

**Figure 1:**
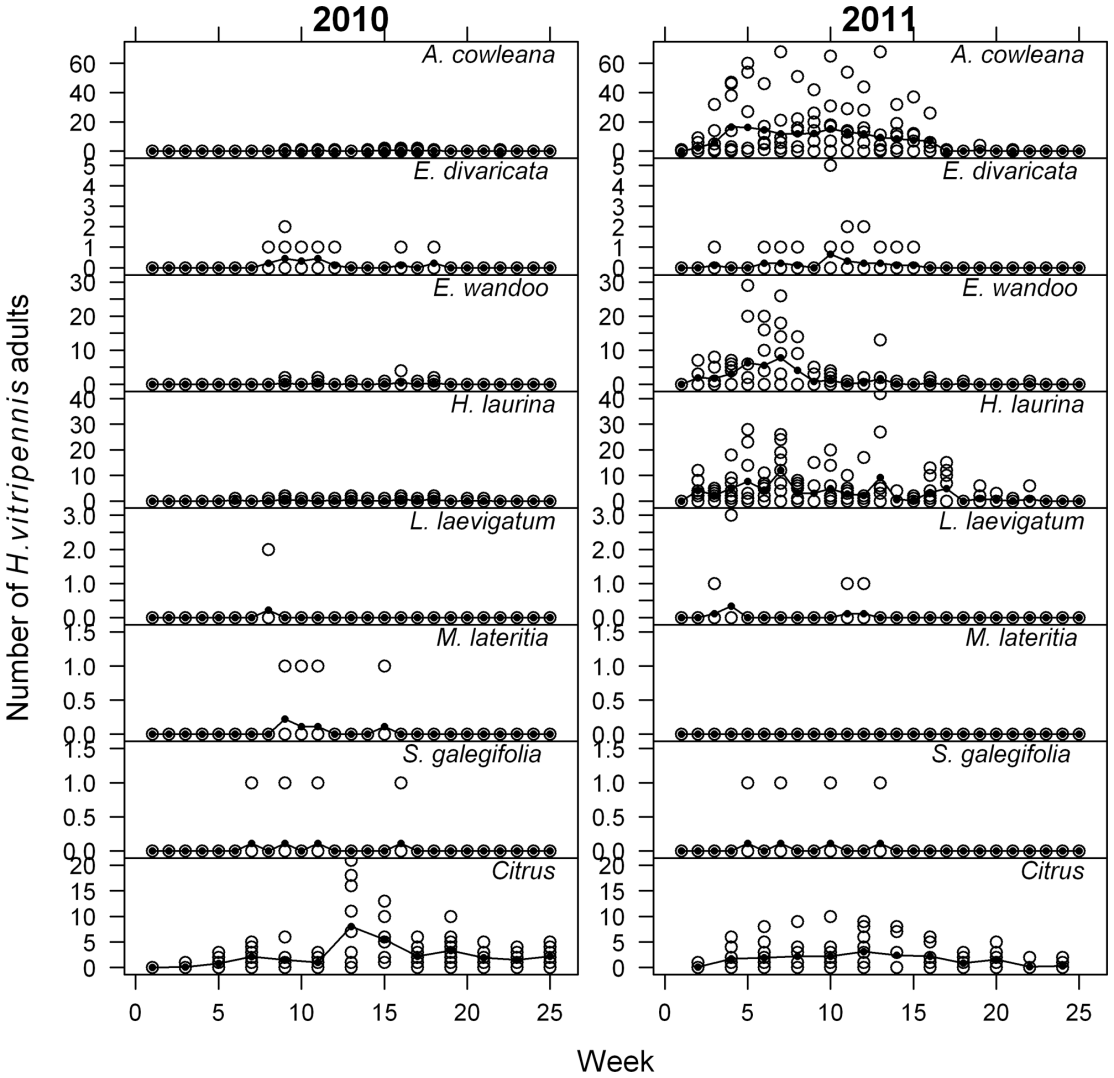
Weekly *Homalodisca vitripennis* adult counts in 2010 and 2011. The raw data is shown as open circles, the means as solid circles. Species with counts of zero are not shown.

Seven of the 12 native Australian plant species (*L. laevigatum*, *A. cowleana*, *E. divaricata*, *E. wandoo*, *H. laurina*, *M. lateritia*, and *S. galegifolia*) were found to be *H. vitripennis* adult feeding hosts in the field. Adult insects were first recorded on study plants on July 7th in 2010 and June 8^th^ 2011 while insects appeared on citrus in June both years. Adult insects were last observed on study plants in October in 2010 and in November in 2011. Adults were observed on citrus until November in both 2010 and 2011. The highest monthly average insect count in 2010 was 4.5 (s.e. = 1.5) which was observed was for citrus in August. However in 2011 the highest monthly average insect counts were observed for *A. cowleana*, *H. laurina* and *E. wandoo* (13.5 s.e. = 3.4, 6.7 s.e. = 1.4 and 5.9 s.e. = 1.4 respectively) exceeding citrus (2.7 s.e. = 0.7). The number of insects observed per week on each plant was generally low with a maximum of four in 2010 (Figure 1). In contrast, 2011 counts were higher with over 60 insects observed on an *A. cowleana* plant for multiple weeks. No clear population spikes were seen in 2010 while two spikes in July and November were recorded for a number of plant species in 2011.

The mean total number of insects recorded on native plants over the 25 week study period was highest for *H. laurina* (2.4) in 2010. The mean of the citrus control plants (30.1) (Table 1) was higher than that of any of the Australian species. In 2011, *A. cowleana* had the highest mean (103.4) (Table 1) of the total number of insects recorded, which far exceeded the figure for citrus (18.80). There was a significant species effect on *H. vitripennis* adult season totals (F_6,54_ = 4.36; p<0.001) with *H. laurina*, *A. cowleana* and *E. divaricata* favoured as hosts in 2010. In 2011, *A. cowleana* had significantly higher adult season total insect counts than all other species (F_6,54_ = 35.56; p<0.001).

**Table 1:** *Homalodisca vitripennis* incidence on Australian native plants in a field survey in California, USA from June–November (n = 9).

Plant species	Adult *Homalodisca vitripennis*		Nymph *Homalodisca vitripennis*		*Homalodisca vitripennis* egg masses	
	2010	2011	2010	2011	2010	2011
	Back transformed Mean (s.e.)	Back transformed Mean (s.e.)	Back transformed Mean (s.e.)	Mean† (s.e.)	Back transformed Mean (s.e.)	Back transformed Mean (s.e.)
*A. cowleana*	1.8 (1.3) a	103.4 (1.4) a	0.1 (1.1) c	0.1 (0.1)	1.6 (1.4) b	5.5 (1.8) cd
*Banksia* #	0	0	0	0	0	0
*C. pulchella*	0	0	0	0	0	2.0 (1.5) d
*C. viminalis*	0	0	0	0	0	0
*E. divaricata*	1.5 (1.2) a	1.8 (1.2) c	1.1 (1.3) ab	3.0 (2.0)	34.3 (1.5) a	51.1 (1.6) a
*E. wandoo*	1.1 (1.3) ab	17.9 (1.5) b	0	0	4.2 (1.5) b	0
*G. lanigera*	0	0	0	0	0	0
*H. laurina*	2.4 (1.3) a	36.8 (1.6) b	1.7 (1.2) a	2.8 (1.6)	26.9 (1.7) a	19.0 (1.7) abc
*L. laevigatum*	0.1 (1.1) c	0.3 (1.2) c	0.5 (1.4) bc	4.7 (2.2)	5.3 (2.0) b	6.6 (2.0) bcd
*M. lateritia*	0.4 (1.2) bc	0	0.2 (1.2) c	0	0	0
*P. ovalifolia*	0	0	0	0	0	0
*S. galegifolia*	0.3 (1.2) bc	0.3 (1.2) c	0.4 (1.2) bc	0	43.6 (1.5) a	12.2 (1.8) bc
Citrus∧	30.1 (4.0)	18.8 (3.4)	8.2 (2.1)	2.1 (0.6)	13.8 (2.5)	15.4 (1.8)

Species back transformed means in each column assigned the same letter are not significantly different; citrus control with mean and standard error (n = 10) also provided.

∧ Citrus plants were not part of the randomised design and data not included in the ANOVA and not transformed.

† Species effect not detected at p = 0.05.

# *Banksia ericifolia×B. spinulosa*.

Six species (*L. laevigatum*, *A. cowleana*, *E. divaricata*, *H. laurina*, *M. lateritia*, and *S. galegifolia*) were found to be *H. vitripennis* nymph hosts in the field (Table 1). Early-stage nymphs (instars 1–3) were first recorded on citrus in June and on study plants in July 2010. The same pattern was seen in 2011. Late stage nymphs (4^th^ and 5^th^ instars) were first observed in citrus in June and Australian plants in July in 2010 and 2011. *H. vitripennis* nymphs were last observed on citrus and the majority of the Australian native plant species in September in both years. The highest monthly average total observed was for citrus in August (9.00 in 2010 and 0.55 in 2011). The plant species effect for the 2010 survey was significant (F_5,46_ = 2.98; p = 0.02) even though the total counts were low (Table 2). In the 2011 survey, the plant species effect was not significant (p = 0.07). A discrepancy was observed between the number of egg masses on a plant and the number of nymphs. There were often many egg masses on a plant yet very few nymphs. No nymphs were observed on sticky cards.

**Table 2:** Percentage of egg masses parasitised and proportional parasitism for each species with s.e., lower and upper 95% confidence intervals (C.I) for 2010 and 2011 showing the total number of leaves with egg masses present (n).

	2010					2011				
Plant species	n	% egg masses parasitised	Proportional parasitism (s.e.)	Lower C.I	Upper C.I	n	% egg masses parasitised	Proportional parasitism (s.e.)	Lower C.I	Upper C.I
*A. cowleana*	4	75	0.72 (0.24)	0.25	1.19	11	73	0.65 (0.14)	0.38	0.92
*Banksia* #	3	67	0.67 (0.33)	0.01	1.32	0	0	0.00	0.00	0.00
*C. pulchella*	7	33	0.30 (1.70)	−0.03	0.63	3	33	0.20 (0.20)	−0.19	0.59
*Citrus*	48	69	0.56 (0.06)	0.43	0.68	30	70	0.64 (0.08)	0.48	0.79
*E. divaricata*	17	88	0.66 (0.09)	0.49	0.83	16	69	0.65 (0.12)	0.42	0.87
*E. wandoo*	20	55	0.46 (0.10)	0.27	0.66	30	57	0.44 (0.08)	0.28	0.60
*H. laurina*	24	42	0.37 (0.09)	0.18	0.55	7	29	0.21 (0.15)	−0.08	0.51
*L. laevigatum*	13	92	0.85 (0.08)	0.69	1.00	15	53	0.44 (0.12)	0.20	0.68
*S. galegifolia*	28	68	0.62 (0.09)	0.44	0.79	12	58	0.58 (0.15)	0.29	0.87

Proportion = number of parasitoids to number of H. vitripennis nymphs.

# *Banksia ericifolia×B. spinulosa*.

Six plant species were found to be *H. vitripennis* oviposition hosts in the field (Table 1). In 2010, egg masses were first observed on citrus and *H. laurina* in June and on the remainder of the Australian native plant hosts in July and last observed on Australian native plant species and citrus in October. In 2011, egg masses were seen on all oviposition hosts in June aside from *C. pulchella* (July) and last seen in August and September. The highest monthly average on a per week basis was observed for *S. galegifolia* in 2010 (9.89) followed by *E. divaricata* (9.50), both in August, while in 2010, the highest monthly average was for *E. divaricata* in July (12.28). The mean of the total number of egg masses recorded on the Australian native plants over the 25 week study period was highest for *S. galegifolia* (43.6) in 2010 and *E. divaricata* (51.1) in 2011, both higher than citrus control plants (Table 1).

The effect of plant species on oviposition was significant in 2010 (F_6,54_ = 7.81, p<0.001). The highest numbers of egg masses were found in *S. galegifolia*, *H. laurina*, and *E. divaricata*, which were significantly higher than the other four species. In 2011, the effect of plant species was also significant (F_6,54_ = 5.39, p<0.001), with increased oviposition observed on *E. divaricata*, and *H. laurina*.

### Parasitism on Australian host plants

All parasitoids that emerged from *H. vitripennis* eggs were identified as *G. ashmeadi*. The highest proportion of parasitoids to *H. vitripennis* nymphs in eggs was seen in *L. laevigatum* in 2010 (0.85) and in *A. cowleana*, *E. divaricata* and Citrus in 2011 (0.65, 0.65 and 0.64 respectively) (Table 2). The percentage of parasitised egg masses was higher in 2010 than in 2011 for all species but Citrus and *E. wandoo*.

### No choice greenhouse feeding study

The effect of species on *H. vitripennis* excreta volume and survival was significant (2010 F_12,205_ = 28.1, p<0.001; 2011 F_12,226_ = 12.7, p<0.001). In 2010, average volumes of excreta produced by insects on *S. galegifolia* and the *V. vinifera* control plants were significantly greater than all other species (8.30, 7.43 ml respectively) followed by insects on *A. cowleana* (3.13 ml) (Table 3). Very low volumes (less than 1 ml) of excreta were produced by insects on all other plant species. In 2011, excreta production was significantly higher on *A. cowleana* (6.70 ml) than on any other species. Insects on *V. vinifera* produced 3.21 ml of excreta over 72 hours, followed by those on *G. lanigera*, *S. galegifolia*, and *H. laurina*. Average excreta production for insects on each of the other species was less than 1 ml (Table 3).

**Table 3:** Effect of Australian native plant species on feeding rate (excreta volume) and predicted survival proportion for 2010 and 2011 for *Homalodisca vitripennis* in a no choice feeding experiment over 72 hours.

	2010		2011	
Plant species	Excreta volume back transformed mean (ml)	Predicted survival proportion (n = 48) back transformed	Excreta volume back transformed mean (ml)	Predicted survival proportion (n = 41) back transformed
*A. cowleana*	3.13 c	0.61 c	6.70 f	0.73 e
*Banksia* #	0.14 a	0.68 cd	1.24 d	0.69 de
*C. pulchella*	0.92 b	0.52 bc	0.19 ab	0.45 abcd
*C. viminalis*	0.42 ab	0.59 bc	0.06 a	0.25 a
*E. divaricata*	0.18 a	0.52 bc	0.17 ab	0.66 de
*E. wandoo*	0.56 ab	0.71 cd	0.76 bcd	0.51 bcde
*G. lanigera*	0.73 ab	0.57 bc	1.85 de	0.56 cde
*H. laurina*	0.66 ab	0.36 ab	1.07 cd	0.32 abc
*L. laevigatum*	0.13 a	0.19 a	0.23 abc	0.30 ab
*M. lateritia*	0.19 a	0.27 a	0.05 a	0.33 abc
*P. ovalifolia*	0.18 a	0.64 cd	0.01 a	0.45 abcd
*S. galegifolia*	8.30 d	0.68 cd	1.60 de	0.56 cde
*V. vinifera*	7.43 d	0.82 d	3.21 e	0.71 e

Species back transformed means in each column assigned the same letter are not significantly different.

# *Banksia ericifolia×B. spinulosa*.

In both years, the effect of plant species on the proportion of *H. vitripennis* that survived to 72 hours in the no choice greenhouse feeding study was significant (2010 F_12,301_ = 4.8, p = <0.001; 2011 F_12,335_ = 3.7, p<0.001). In 2010, the highest predicted survival was for insects on the *V. vinifera* control plants (0.82), *E. wandoo*, *Banksia hybrid*, *S. galegifolia* and *P. ovalifolia* (0.71, 0.68, 0.68 and 0.64 respectively) (Table 3). Highest predicted *H. vitripennis* survival after 72 hours in 2011 was for *A. cowleana* (0.73), *V. Vinifera* (0.71), *Banksia hybrid* (0.69), *E. divaricata* (0.66), *G. lanigera* (0.56) and *E. wandoo* (0.51) (Table 3).

### Nymph development greenhouse study

Four plant species and *V. vinifera* plants allowed development of nymphs to adults. *Acacia cowleana*, *G. lanigera* and *S. galegifolia* produced adult *H. vitripennis* in approximately 40 days. Nymphs on *L. laevigatum* developed into adults in approximately 70 days, which was a significantly longer development period than the other Australian plant species and *V. vinifera*. *Acacia cowleana* had a 63% mortality rate, *G. lanigera* and *L. laevigatum* had a 92% mortality rate, *S. galegifolia* had a 67% mortality rate and the grapevine controls had an 88% mortality rate. Nymph mortality rates were 100% for all other Australian native plant species.

Mortality occurred for 2^nd^, 3^rd^ and 4^th^ instar nymphs and the majority of nymphs that died survived until the 2^nd^ instar stage only (Table 4). No mortality was recorded in 1^st^ or 5^th^ instar nymphs on any plant species. The highest mean number of days until death was recorded for *H. laurina* followed by *E. divaricata*. The lowest mean number of days until death was seen in *L. laevigatum*, *C. viminalis* and *A. cowleana* (<10 days).

**Table 4:** Life history responses of *H. vitripennis* to Australian native plants (combined 2010 and 2011 data).

Plant species	1^st^ instar	2^nd^ instar	3^rd^ instar	4^th^ instar	5^th^ instar	Mean no. of days until nymph death (s.e.)	Proportion reaching adulthood	Mean no. days until adulthood (s.e.)
*A. cowleana*	0	2.8	0	0	0	6.0, n = 1, (-)	0.25	40.6, n = 9, (2.88)
*Banksia* #	0	13.9	8.3	0	0	19.4, n = 8, (4.28)	0	-
*C. pulchella*	0	2.8	2.8	2.8	0	24.3, n = 3, (11.86)	0	-
*C. viminalis*	0	2.8	0	0	0	6.0, n = 1, (-)	0	-
*E. divaricata*	0	13.9	5.6	0	0	27.8, n = 7, (6.03)	0	-
*E. wandoo*	0	13.9	11.1	0	0	13.2, n = 9, (1.96)	0	-
*G. lanigera*	0	2.8	0	2.8	0	31.0, n = 2, (8.00)	0.06	37.5, n = 2, (2.50)
*H. laurina*	0	8.3	5.6	5.6	0	28.9, n = 7, (7.08)	0	-
*L. laevigatum*	0	8.3	0	0	0	41.3, n = 3, (0.67)	0.06	70.5, n = 2, (1.50)
*M. laterita*	0	16.7	0	0	0	5.7, n = 3, (3.16)	0	-
*P. ovalifolia*	0	5.6	0	0	0	21.5, n = 6, (6.93)	0	-
*S. galegifolia*	0	2.8	5.6	0	0	17.0, n = 2, (8.20)	0.22	39.6, n = 8, (1.45)
*V. vinifera*	0	11.1	0	0	0	13.3, n = 4, (2.39)	0.08	45.3, n = 3, (2.19)

The instar figures are the proportion of nymphs that died at each instar stage out of 36; the remainder either survived or were eaten by ants. n is the total number of nymphs for which there was a record of date of death or total number of adults.

# *Banksia ericifolia×B. spinulosa*.

## Discussion

The choice and no-choice field and greenhouse studies presented give an indication of the ability of Australian native plants to support feeding, oviposition, and nymph development of *H. vitripennis* and *H. vitripennis* egg parasitism by mymarid egg parasitoids in the field. Of the Australian native plants tested here, *A. cowleana* was the most favoured adult *H. vitripennis* feeding host followed by *H. laurina* and *E. wandoo*. These are all large-leaved species with easily accessible sections of unobscured stem material and prominent veins in the leaves. These factors may have favoured their selection as feeding hosts. The plants on which *H. vitripennis* were never observed feeding, in contrast, characteristically had a compact, shrubby form with small, tightly packed leaves which may have discouraged feeding. Future work is required to test such plant morphology-related hypotheses because data on plant characters was not collected in the present study. Insect numbers observed in 2011 were much greater than 2010, especially on *A. cowleana*. Adult numbers were also higher on citrus and it is possible that the *H. vitripennis* population was higher in 2011 due to increased reproduction, reduced parasitism and predation, or both. Laboratory parasitism results support the theory of reduced parasitoid pressure in the second year with the proportion of uneclosed parasitoids to *H. vitripennis* in *H. vitripennis* egg masses being lower in 2010 than 2011. Krugner *et al.* [25] argue that Argentine ants may play a role in *H. vitripennis* population regulation in citrus orchards and perhaps ant numbers were higher in 2010 than 2011 but it is also possible that the Australian hosts were favoured more in 2011 due to improving vigour because of increasing plant age and better establishment.

*Homalodisca vitripennis* adult populations on Australian native host plants followed a similar pattern to that seen on citrus. Although temperatures were similar in both years, in 2010, no clear population spikes were seen, while two clear spikes in July and November were recorded for a number of the Australian species in 2011. The 2011 pattern has been recorded previously in Riverside [26], [27] and is likely a manifestation of the two generations per year typical of Californian *H. vitripennis* populations.

When tested under no choice conditions in the greenhouse, *H. vitripennis* had a much broader feeding host range than that observed in the field. This points to a major advantage of the realism in testing plants under choice conditions in the field; something that is not possible in a quarantine laboratory and an indication of the value of the present approach of testing an invasive species in its invasive range using potential hosts in their established exotic range. It appears that under greenhouse conditions *C. viminalis*, *H. laurina* and *L. laevigatum* are not suitable feeding hosts for *H. vitripennis* adults as the majority of insects did not feed and died, whilst those that did feed in small amounts also died, indicating that the xylem fluid may not contain enough nutrition for survival at the time these studies were conducted. It is also possible that the xylem tension of these plants was not conducive to feeding, physical access to sap was hindered, or that the insects never located the food source [10], [16]. The deviation from the field study findings for *H. laurina* may be because adults are attracted by visual or chemical cues from these plants but feeding cues or xylem chemistry do not align with the insect's needs. A background level of natural mortality was expected and this level was indicated by the grapevine controls which had an average mortality rate of 25%, a rate that was lower than that seen on any of the Australian native species.

In 2010, *S. galegifolia* and *A. cowleana*, had significantly higher feeding rates compared to all other Australian native plant species with *A. cowleana* exhibiting this also in 2011. Both are nitrogen fixing plants (Fabaceae). For this reason, it is possible that the xylem of *S. galegifolia* and *A. cowleana* had a higher nutritional value than that of the other plant species, resulting in increased feeding rates. It is also possible that plant transpiration rates and xylem tension played a role in the different feeding rates. *H. vitripennis* feeding tends to be higher with decreased xylem tension in grapevines [16].

Nymphs were observed in the field on five of the 12 plant species as well as the citrus controls, four of which were also found to be adult feeding and oviposition hosts. Nymph numbers were low (<10 nymphs per plant), however, this may be a function of the nymphs' small size and inconspicuous colouring making them difficult to see. The discrepancy between the number of egg masses on a plant and the low number of nymphs could be due to predation or the nymphs may have been leaving the plants in search of a more favoured feeding host. Nymphs may have jumped further than the sticky cards or the Vaseline® may have been too weak under warm temperature to hold insects, accounting for the lack of nymphs trapped. The majority of 3^rd^ instar nymphs in a study by Tipping *et al.* (2004) jumped less than 40 cm yet 5^th^ instar nymphs can potentially leap just over 1 m [28]. In the current experiment, travel to another host plant seems unlikely because the closest plant was over 2 m away and nymphs would have had to cross bare earth under hot, dry summer conditions to reach it. However, there was one record of nymphs on *M. lateritia*, a species that was not recorded as an oviposition host so some evidence exists to support nymph host switching via jumping or walking. If nymphs stayed on the plant but it was simply not providing the required nutrition for survival it is expected that dead nymphs would have fallen from branches onto the sticky cards below. As no nymphs were recovered from sticky cards to indicate death or independent nymph movement away from the plant and mymarid parasitoids kill eggs before hatching, it is most likely that the high parasitism rates (over 50% all species except *C. pulchella* and *H. laurina*) account for the lack of nymphs seen. Predation is also a possibility although no predators were observed on the plants or sticky traps during survey periods.

In the no choice greenhouse setting, nymphs survived to adulthood on *A. cowleana*, *G. lanigera*, *L. laevigatum* and *S. galegifolia*. These species were shown to be oviposition hosts in the field supporting the theory that adult insects oviposit on host plants that are nutritionally suitable for nymph development [13], [14]. It is difficult to gain full nutrition for development on one host plant due to varied nutritional requirements across different instars. For this reason, 100% mortality was observed on eight of the 12 Australian plant species tested, and even those plants that could support development had high mortality rates. Previous studies have reported mortality rates to adulthood ranging from 100% [13] to 17.5% [29] depending on the host plant species.

Host plant determines the period of development from first instar nymph to adult emergence for *H. vitripennis*. On sunflower (*Helianthus annuus* L.) this timeframe is approximately 35 days, on chrysanthemum (*Chrysanthemum morifolium* L.) 42 days and on *Euonymus japonica* Thunb. 87 days [29]. Although not directly comparable due to differing temperature and plant fertilisation regimes, these figures are similar to development times seen on the Australian native plants. *Homalodisca vitripennis* on *L. laevigatum* took an average of 70.5 days to emerge as adults which is significantly longer than the grape control. In contrast, *A. cowleana*, *G. lanigera* and *S. galegifolia* gave developmental times similar to that on the grape control. *Acacia cowleana* and *S. galegifolia* also gave the lowest mortality rates indicating that these two species may be important *H. vitripennis* developmental hosts in an Australian setting.

*Homalodisca vitripennis* oviposited on a broader range of Australian native species than they fed or completed nymph development on (nine of the 12 species). The highest number of egg masses was observed on *E. divaricata*. Typically egg masses are oviposited into the abaxial leaf surface yet tended to be located on the upper surface of the leaves on *H. laurina* and *A. cowleana* which is unusual. It is possible that these two species have adaptations (perhaps xerophytic) that make the upper surface easier to oviposit into such as a thicker cuticle or fewer trichomes. *Homalodisca vitripennis* has been shown to prefer cv Eureka lemons trees over cv Lisbon lemon trees for oviposition in greenhouse studies which is thought to be due to leaf thickness and morphology [30]. *Prostanthera ovalifolia* is commonly known as ‘mint bush’ due to its strong aroma. Perhaps the volatile compounds responsible for this odor are an effective deterrent to *H. vitripennis* or it masks kairomones thereby altering attraction cues and preventing oviposition on this species. Parasitism was not observed on Australian native plants or citrus control plants until July, just after egg masses had begun to appear. This is likely to be due to a lag time between *H. vitripennis* and natural enemy population peaks. Parasitoids require time to build up population levels following the seasonal peak in *H. vitripennis* numbers (D Morgan 2010 pers. comm.). Over winter, prior to the spike in *H. vitripennis* numbers, there were very few egg masses for parasitoids to utilize therefore a high natural enemy population cannot be sustained year round.

*Gonatocerus ashmeadi* is the most common species of parasitoid in southern California attacking *H. vitripennis* eggs [31] which explains why this was the only species that emerged from collected eggs. The signals that attract parasitoids to *H. vitripennis* eggs are not fully understood but are thought to be volatiles that are released as part of the plants' secondary defence mechanism [19]. If this is the case, the observation that egg masses on Australian native plants had high levels of parasitism implies that an evolutionary history with the pest is not necessary to develop and induce these defences. Plant species that gave lower parasitism rates such as *H. laurina* may act as a refuge to some extent where *H. vitripennis* egg masses are less likely to be parasitised.

The Australian plant species tested here have been categorised based on their suitability as *H. vitripennis* hosts (Table 5). Those species in category A are considered to be important in aiding an invasion as they have been shown to be feeding, nymph development, and oviposition hosts in the field. Category B indicates plants that supported at least one of the three life stages (egg, nymph or adult) in the field, while category C indicates species that were not shown to support any *H. vitripennis* life stage in the field. This does not mean they are not hosts, however, they are likely to play a small, if any, role in an incursion where *H. vitripennis* has access to a range of native host plants.

**Table 5:** *Homalodisca vitripennis* host status of Australian native plants in the field.

Species	Adult host	Nymph host	Oviposition host	Risk category
*A. cowleana*	X	X	X	A
*Banksia* #	-	-	-	C
*C. pulchella*	-	-	X	B
*C. viminalis*	-	-	-	C
*E. divaricata*	X	X	X	A
*E. wandoo*	X	-	X	B
*G. lanigera*	-	-	-	C
*H. laurina*	X	X	X	A
*L. laevigatum*	X	X	X	A
*M. lateritia*	X	X	-	B
*S. galegifolia*	X	X	X	A
*Citrus*	X	X	X	A

# *Banksia ericifolia×B. spinulosa*.

## Conclusion

The adopted approach of studying the invading species *in situ* and assessing the response of host plant species in their exotic location offers benefits as it removes the need for expensive quarantine facilities and administrative clearance for the importation of the species of interest. It is therefore a valuable approach for future research in biosecurity risk assessment and invasive species- host interactions. Past incursion case studies indicate that invasions are likely to occur or be detected first in urban areas [32] that have a very high diversity of plants – native and exotic (e.g. commercial citrus varieties in Australia). These findings have implications for both preventative surveillance and management of vegetation in the event of an incursion in Australia. It would be beneficial for those species shown to be *H. vitripennis* hosts to be included in all monitoring activities to detect an incursion early. *Homalodisca vitripennis* host choice also plays a role in *X. fastidiosa* risk to native Australian plants with high or low feeding preference greatly influencing likelihood of inoculation by insects carrying the pathogen. There is a potential role that could be played by 1) nursery regulation in terms of buying and selling native plant species that are shown to be hosts and shipping them throughout the country and 2) landscape management [33], for example removing ‘high risk’ species from vegetation on the margins of susceptible crops in the event of an incursion and replacing them with species that are not shown to be hosts in order to help prevent the establishment of *H. vitripennis*. However, this management option would require tight regulatory restrictions to ensure best practice and prevent fragile landscapes and ecosystems from being altered unnecessarily.
